# Usefulness of dynamic regression time series models for studying the relationship between antimicrobial consumption and bacterial antimicrobial resistance in hospitals: a systematic review

**DOI:** 10.1186/s13756-023-01302-3

**Published:** 2023-09-12

**Authors:** Paul Laffont-Lozes, Romaric Larcher, Florian Salipante, Geraldine Leguelinel-Blache, Catherine Dunyach-Remy, Jean-Philippe Lavigne, Albert Sotto, Paul Loubet

**Affiliations:** 1 Department of Pharmacy, Nimes University Hospital, Nimes, France; 2 Infectious and Tropical Diseases Department, Nimes University Hospital, Nimes, France; 3 PhyMedExp, INSERM U1046, CNRS, University of Montpellier, Montpellier, France; 4 Department of Biostatistics, Epidemiology, Public Health, and Innovation in Methodology (BESPIM), University of Montpellier, Nîmes University Hospital, Nimes, France; 5 Department of Microbiology and Hospital Hygiene, Nimes University Hospital, Nimes, France; 6 VBIC, INSERM U1047, University of Montpellier, Nimes, France; 7 Service des Maladies Infectieuses et Tropicales, Hôpital Caremeau – CHU de Nimes, 1 Place Robert Debre, Nîmes, 30000 France

**Keywords:** Antimicrobial, Dynamic regression, Healthcare-associated infections, Resistance, Time series analysis

## Abstract

**Backgroung:**

Antimicrobial resistance (AMR) is on the rise worldwide. Tools such as dynamic regression (DR) models can correlate antimicrobial consumption (AMC) with AMR and predict future trends to help implement antimicrobial stewardship programs (ASPs).

**Main body:**

We carried out a systematic review of the literature up to 2023/05/31, searching in PubMed, ScienceDirect and Web of Science. We screened 641 articles and finally included 28 studies using a DR model to study the correlation between AMC and AMR at a hospital scale, published in English or French. Country, bacterial species, type of sampling, antimicrobials, study duration and correlations between AMC and AMR were collected. The use of β-lactams was correlated with cephalosporin resistance, especially in *Pseudomonas aeruginosa* and Enterobacterales. Carbapenem consumption was correlated with carbapenem resistance, particularly in *Pseudomonas aeruginosa, Klebsiella pneumoniae* and *Acinetobacter baumannii.* Fluoroquinolone use was correlated with fluoroquinolone resistance in Gram-negative bacilli and methicillin resistance in *Staphylococcus aureus*. Multivariate DR models highlited that AMC explained from 19 to 96% of AMR variation, with a lag time between AMC and AMR variation of 2 to 4 months. Few studies have investigated the predictive capacity of DR models, which appear to be limited.

**Conclusion:**

Despite their statistical robustness, DR models are not widely used. They confirmed the important role of fluoroquinolones, cephalosporins and carbapenems in the emergence of AMR. However, further studies are needed to assess their predictive capacity and usefulness for ASPs.

**Supplementary Information:**

The online version contains supplementary material available at 10.1186/s13756-023-01302-3.

## Background

Antimicrobial resistance (AMR) is increasing worldwide [1, 2] and by 2050, 10 million deaths per year could be related to infections caused by multidrug-resistant (MDR) bacteria, surpassing cancers [3]. Given the close link between antimicrobial consumption (AMC) and AMR, the World Health Organization (WHO) has encouraged antimicrobial stewardship programs (ASPs) and AMR surveillance in response to this alarming situation [4–6].

Observational or quasi-experimental studies using time series models are recognized as the gold standard for estimating the correlation between AMC and AMR [7–9]. Among these models, dynamic regression (DR) models, originally used in economics to study stock market fluctuations, are probably one of the best options for studying correlations between AMC and AMR [10].

Combining the advantages of Box and Jenkins’ autoregressive integrated moving average (ARIMA) [11] and Pankratz’s linear transfer function (LTF) [12], DR models can take into account the time lag between antimicrobial use and the emergence of AMR, as well as the prior prevalence of AMR, to best estimate the correlation between AMR and AMC. In addition, multivariate DR models can assess the effect of the use of different antimicrobials on AMR, and the burden of their use in relation to other mechanisms involved in the emergence of AMR [13].

Finally, DR models could be used to evaluate existing ASPs and to develop new ones, targeting the consumption of antibiotics that are more closely linked to the emergence of AMR [14]. However, their predictive capacity is the subject of debate and could limit their use to retrospective analyses only [15]. Indeed, external validation of DR models has been performed in few studies [15], in which they seems to be much better at describing the link between prior AMC and AMR, rather than predicting the emergence of resistance on the basis of presumed AMC.

In the last decade, numerous studies have been published, and it is not easy for readers to have an overview of these results, which can nevertheless influence our practice and the choice of antimicrobial therapy, particularly in hospital setting.

This systematic review aims to summarize the correlations between AMC and AMR reported in studies using DR models, and to explore the predictive ability of these models for use in the assessment and construction of ASPs.

## Main text

### Methods

#### Search strategy

The methodology of this literature review followed the updated Preferred Reporting Items for Systematic Reviews and Meta-Analyses (PRISMA) recommendations [16]. The study was registered on PROSPERO (CRD42022324469), the international prospective register of systematic reviews. Articles included in this systematic review were obtained from three databases of peer-reviewed literature: PubMed (NLM database), ScienceDirect and Web of Science. PubMed and Web of Science databases were searched using the following terms: (“antibiotic resistance” OR “antimicrobial resistance”) AND (“antibiotic consumption” OR “antimicrobial consumption” OR “antibiotic use” OR “antimicrobial use” OR “stewardship program” OR “intervention”) AND (“time series analysis” OR “times-series” OR “dynamic regression” OR “autoregressive integrated moving average” OR “ARIMA” OR “linear transfer function” OR “LTF”). ScienceDirect database was searched using the following terms: (“antibiotic” OR “antimicrobial) AND (“resistance”) AND (“consumption” OR “utilization” OR “use”) AND (“dynamic regression” OR “autoregressive integrated moving average” OR “ARIMA”). Database was searched from 2000 to 2023 (“2000/01/01” (date—publication): “2023/05/31” (date—publication)). Finally, we searched in the reference lists of selected articles to find additional studies.

#### Inclusion/exclusion criteria

All studies that used DR (or analogous) models and examined the in-hospital correlation between AMC and AMR rates or incidences, regardless of bacterial species, were eligible for inclusion. DR models are identified with different terminology [17], thus a statistician familiar with DR models (F.S.) checked all the models. DR models can be either univariate studying each antimicrobial separately, or multivariate studying several antimicrobials simultaneously. Community-based studies or those assessing both the hospital and community setting were not eligible for inclusion. Studies using other time series models (such as interrupted time series) were not included. Articles were limited to the English or French language. Reviews and animal studies were excluded. Meta-analyses with heterogeneous studies (different bacteria, different antimicrobials), were also ruled out.

#### Study selection

Two independent reviewers (P.L.-L. and A.S.) blindly screened potentially eligible abstracts. Then, full-text articles were assessed by two reviewers (P.L.-L. and F.S.). Whenever a discrepancy between reviewers arose after full-text reading, study’s eligibility was discussed with another reviewer (A.S.) until a consensus was achieved.

#### Data collection

Author names, year of publication, study period and duration, study location, microorganisms, sample types, antimicrobial treatments and study duration were collected from each report. When calculated, the coefficient of determination (R^2^), which represented the percentage of variations in AMR explained by the DR as a function of AMC, was recorded. The authors of studies were contacted in case of missing or incomplete data. We did not carry out a meta-analysis due to the heterogeneity of the studies examined (different bacteria and different antibiotics analyzed), which would have led to inconclusive results.

#### Quality of studies

Conventional tools for assessing the risk of bias in non-randomized studies (EPOC, ROBINS, ORION reports…) were not suitable to assess the quality of quasi-experimental epidemiological time-series studies. Thus, after consultation with our team of methodologists and research of relevant existing tools, we created a specific checklist (Table S1) adapted from those of the Quebec university Hospital [18], the Newcastle-Ottawa Scale [19] and the STROBE checklist [20]. The quality of the studies was independently assessed by two reviewers (P.L.-L. and F.S.). Discrepancies were resolved by discussion with another reviewer (A.S.) to reach a consensus.

## Results

### Search results

**Fig. 1:**
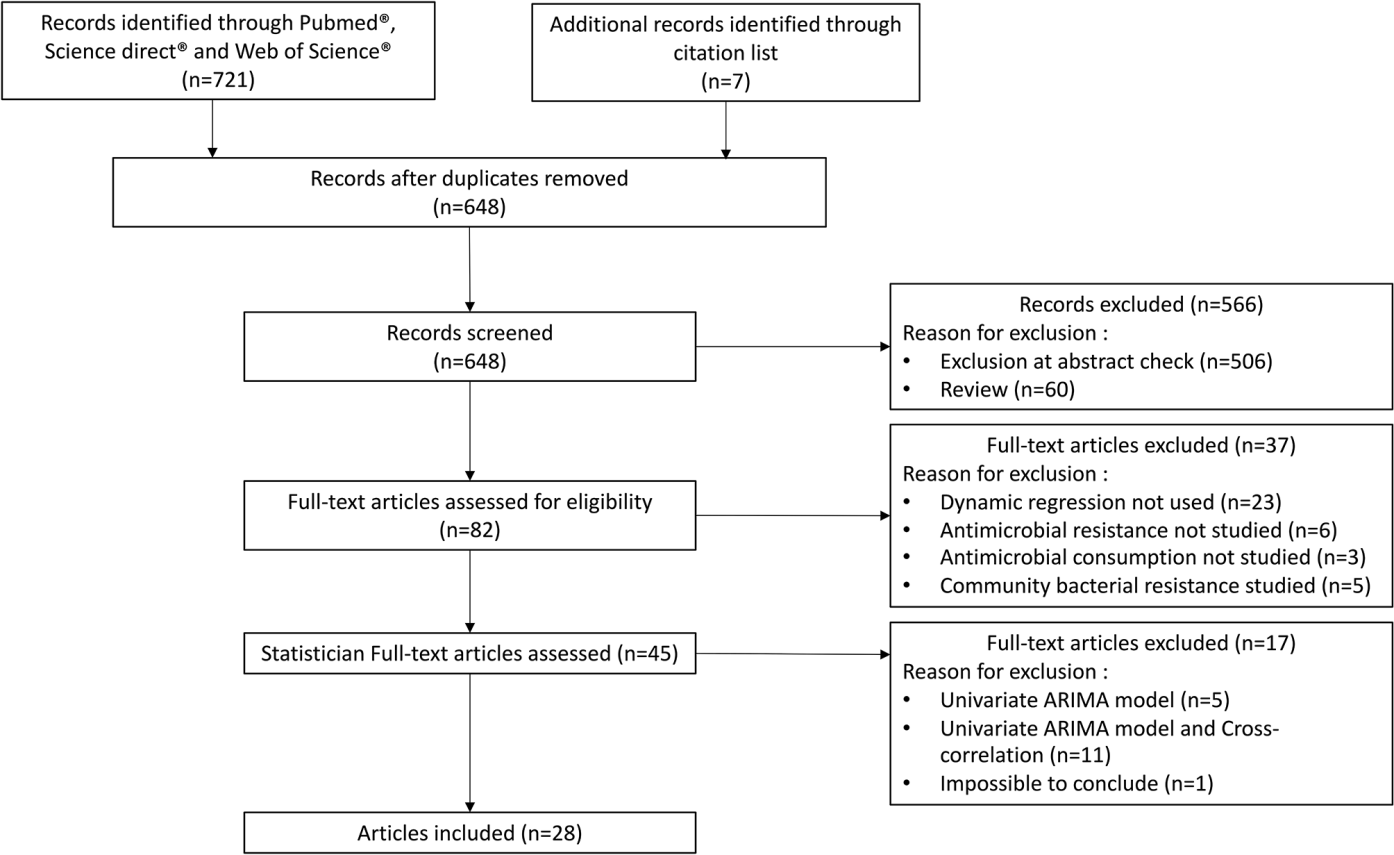
Study selection flow chart

The database search yielded 641 articles and seven additional records were identified from reference lists. Of these, 566 were excluded after abstract screening and 37 were excluded after full-text review. A further 17 articles were excluded after analysis for statistical issues (including 11 studies using ARIMA model and cross-correlation analysis without DR, and one study in which DR use was not confirmed despite attempts to contact the authors). Finally, 28 studies [8, 13–15, 21–44] met the inclusion criteria (Table 1; Fig. 1).

**Table 1:** Characteristics of the studies assessing the correlation between the antimicrobial consumption and bacterial antimicrobial resistance using dynamic regression models

Reference	Country	Duration	Bacteria species studied	Antimicrobial studied	Sample type	AMR / AMC correlated (lag)	R² of the model
Aldeyab 2008	UK	60 months	*Staphylococcus aureus*	All classes of antimicrobials	All types of samples from patients in the unit for > 48 hours, with deduplication + nasopharyngeal screening samples	MRSA / Macrolides (4 months), Fluoroquinolones (1 month)^1^, 3rd generation cephalosporins (2 months) and Amoxicillin – Clavulanic Acid (1 month)	78% ^2^
Bertrand 2012	France	108 months	*Staphylococcus aureus*	All classes of antimicrobials	All types of samples from patients in the unit for > 48h or positive in the previous 12 months with deduplication	MRSA / Macrolides (2 months), Fluoroquinolones (2 months), Aminoglycoside (1 month)	41%^2^
Conlon-birgham 2019	UK	72 months	*Staphylococcus aureus*	All classes of antimicrobials	All types of samples from patients in the unit for > 48 hours, with deduplication of following 7 days samples + nasopharyngeal screening samples	MRSA / Macrolides (1 month), Fluoroquinolones (3 months) and Piperacillin-Tazobactam (1 month)	UC
Erdeljić 2011	Croatia	15 months	*Pseudomonas aeruginosa*	Piperacillin-Tazobactam, Ciprofloxacin and Carbapenems	All types of samples	Resistance to Ciprofloxacin / Ciprofloxacin (1month) Resistance to Meropenem / Meropenem (0 months) Resistance to Cefepime / Cefepime (2 months) *P.aeruginosa* MDR / Meropenem (0 months)	UC
Gharbi 2015	UK	72 months	*Klebsiella pneumoniae*	Meropenem	All types of samples + rectal screening sample	*K. pneumoniae* OXA-48 / Meropenem (1 year)	79%
Hocquet 2008	France	84 months	*Pseudomonas aeruginosa*	Aminoglycosides, Fluoroquinolones, Cephalosporins, Penicillins, Carbapenems	All types of samples with deduplication	*P. aeruginosa* displaying MexXY-OprM overproduction / aminoglycoside (0,3,4,6 months) fluoroquinolone (0,5 and 6 months) and antipseudomonal cephalosporin (2 months) *P. aeruginosa* displaying MexXY-OprM overproduction (negative correlation) / non-antipseudomonal cephalosporin (0,2 and months), penicillin (0,1,3 and 5 months) and carbapenem (0,2,5 and 6 months)	81%
Hsueh 2005	Taiwan	13 years	*Escherichia coli* *Pseudomonas aeruginosa*	Cefotaxime, ceftazidime	All types of samples with deduplication in the following 7 days	Cefotaxime resistant *E.coli /* cefotaxime (NS) Ceftazidime resistant *P.aeruginosa* / Ceftazidime (NS)	UC
Kaier 2009	Germany	34 months	*Escherichia coli*, *Enterobacter Cloacae, Klebsiella sp. Acinetobacter sp., Citrobacter sp.*	All classes of antimicrobials	All types of samples from patients in the unit for > 48 hours, with deduplication + rectal screening sample	ESBL / 3rd generation Cephalosporins (3 months), Fluoroquinolones (1 month)	75%^2^
Kaier 2009	Germany	58 months	*Staphylococcus aureus*	All classes of antimicrobials	All types of samples from patients in the unit for > 48 hours, with deduplication	MRSA /2nd generation Cephalosporins (1 month), 3rd generation Cephalosporins (3–4 months), Fluoroquinolones (4 month), Lincosamides (2 months)	66%^2^
Kousovista 2021	Greece	48 months	*Acinetobacter baumannii*	Cephalosporins, Fluoroquinolones, Meropenem, Colistin and Piperacillin-Tazobactam	All types of samples with deduplication	Resistance to Meropenem / Meropenem (2 month), Resistance to Ciprofloxacin / Ciprofloxacin (1 month), Resistance to Cefepime / Cefepime (2 months)	63% 66% 62%
Kritsotakis 2008	Greece	84 months	*Enterococcus sp.*	Amoxicillin-Clavulanic acid, Piperacillin-Tazobactam, Cephalosporins, Fluoroquinolones, Carbapenems, Glycopeptides, Metronidazole, Clindamycin	All types of samples with deduplication + rectal screening sample	ERV / glycopeptides (1 month), 3rd generation Cephalosporins (1 month), Fluoroquinolones (2 months) ERV (negative correlation) / Amoxicillin- Clavulanic Acid (6 months)	56%
Laffont-Lozes 2023	France	72 months	*Escherichia coli*	Penicillins, Amoxicillin-Clavulanic Acid, Piperacillin-Tazobactam, Cephalosporins, Fluoroquinolones, Carbapenems, Aminoglycosides, Sulfonamides	All types of samples from patients in the unit for > 48h or positive in the previous 1 months with deduplication	Resistance to Fluoroquinolones / Fluoroquinolones (0 to 5 months), Piperacillin-Tazobactam (0 to 3 months) Amoxicillin-Clavulanic Acid resistance / Amoxicillin-Clavulanic Acid (0 to 1 months) Penicillins resistance / 3rd generation Cephalosporins (5 and 8 months), Amoxicillin-Clavulanic Acid (0 and 5 months), Carbapenem (0 month) Cephalosporins resistance / 4rd generation Cephalosporins (0 month), Piperacillin-Tazobactam (0 to 2 months), Fluoroquinolones (0 to 4 months)	54% 53% 36% 15%
Lee 2012	Taiwan	84 months	*Staphylococcus aureus*	All classes of antimicrobials	All types of samples with deduplication	MRSA / penicillins, including b-lactamase inhibitors (1 month)	26%^2^
Lepper 2002	Germany	48 months	*Pseudomonas aeruginosa*	Imipenem, Cephalosporins, Aminoglycosides, Ciprofloxacin, Piperacillin-Tazobactam	All types of samples	Resistance to Ceftazidime / Imipenem (0 and 1 month) Resistance to Piperacillin-tazobactam / Imipenem (0 and 1 month), Resistance to imipenem / Imipenem (0 and 1 month)	UC
López-Lozano 2000	Spain	90 months	*Pseudomonas aeruginosa, Escherichia coli, Proteus sp., Klebsiella sp., Enterobacter sp.*	Ceftazidime and Imipenem	All types of samples with deduplication	Gram-negative bacilli Resistant to 3rd generation Cephalosporins / Ceftazidime (1 month) *Pseudomonas aeruginosa* resistant to Imipenem / Imipenem (1 month)	44% 63%
Mahamat 2005	France	84 months	*Escherichia coli*	Fluoroquinolones	Cytobacteriological urine examination with deduplication	Resistance to Ofloxacin / Ofloxacin (4 months), Ciprofloxacin (4 months) Resistance to Ciprofloxacin / Ciprofloxacin (4 months), Ofloxacin (4 months)	64% 40%
Mahamat 2007	UK	96 months	*Staphylococcus aureus*	All classes of antimicrobials	All types of samples with deduplication	MRSA / Macrolides (5 months), Fluoroquinolones (2 months)	UC
Monnet 2004	UK	55 months	*Staphylococcus aureus*	All classes of antimicrobials	All types of samples except for nasopharyngeal screening samples	MRSA / 3rd generation Cephalosporins (4–7 months), Macrolides (1–3 months), Fluoroquinolones (4–5 months)	90%
O’Riordan 2022	Ireland	16 quarters	Enterobacterales and *Enterococcus faecium*	ceftriaxone, ciprofloxacin, levofloxacin, ertapenem, meropenem, piperacillin-tazobactam, gentamicin, co-trimoxazole and aztreonam; vancomycin	All types of samples with deduplication	*E.coli* 3rd generation Cephalosporins resistant (negative correlation) / Piperacillin-tazobactam (0 and 1 quarter)	86%
Ortiz-Brizuela 2020	Mexico	66 months	Enterobacterales	All classes of antimicrobials	All types of samples with deduplication	Resistance to Carbapenem non-susceptible Enterobacterales / Piperacillin- Tazobactam (6 months) Resistance to carbapenemase-producing Enterobacteriaceae/ Piperacillin- Tazobactam (6 months) Resistance to OXA-232 carbapenemase-producing Enterobacteriaceae / Piperacillin- Tazobactam (6 months)	21% 19% 24%
Qu 2019	China	60 months	*Klebsiella pneumoniae*	Doxycycline, Cephalosporins, Carbapenems	All types of samples with deduplication	Resistance to Carbapenems / Cephalosporins (0 and 1 quarter), Meropenem (0 and 1 quarter), Doxycyclin (2 quarters)	46–94% (according to antibiotic studied)
Tansarli 2018	Greece	174 months	*Klebsiella pneumoniae*	Colistin	Blood culture	Resistance to Colistin / Colistin (1 quarter)	69%
Tóth 2019	Hungary	142 months	*Escherichia coli, Klebsiella pneumoniae, Klebsiella oxytoca, Pseudomonas aeruginosa, Acinetobacter baumannii*	Cephalosporins, Carbapenems, Colistin	All types of samples, with deduplication during the following month	*E.coli*: Resistance to Cephalosporins / Cephalosporins (1 month and 4 months) *Klebsiella pneumoniae*: Resistance to Cephalosporins/Cephalosporins (4 months and 5 months) Resistance to Carbapenems/Carbapenems (6 months) *Pseudomonas aeruginosa*: Resistance to Cephalosporins/Cephalosporins (0 months) Resistance to Carbapenems/Carbapenems (0 months and 1 month) *Acinetobacter baumannii*: Resistance to Carbapenems/Carbapenems (4 months)	UC
Vernaz 2008	Switzerland	80 months	*Staphylococcus aureus*	All classes of antimicrobials	All types of samples with deduplication	MRSA / Fluoroquinolones (1 month), Macrolides (1 and 4 month), Cephalosporins (3 to 5 month), Piperacillin-Tazobactam (3 month)	57%^2^
Vibet 2015	France	84 months	Enterobacterales	All classes of antimicrobials	All types of samples from patients in the unit for > 48 hours, with deduplication	ESBL / 3rd and 4th-generation cephalosporins (5 months), Fluoroquinolones (3 months), Lincosamides (1 months), other antibacterial agents (4 months), Tetracyclines (3 months) ESBL (negative correlation) / Nitrofurantoin (1month), Ticarcillin and piperacillin with or without enzyme inhibitor (4 month)	UC
Wang 2021	China	90 months	*Klebsiella pneumoniae*	Penicillins, Penicillins + β-lactamases inhibitor, Cephalosporins, Carbapenems, Aminoglycosides, Fluoroquinolones, Sulfonamides	All types of samples with deduplication	Resistance to Amikacin / Amikacin (0 months) Resistance to Ciprofloxacin / Ciprofloxacin (0 months)	45% 62%
Willmann 2013	Germany	120 months	*Pseudomonas aeruginosa*	Carbapenems, Cephalosporins, Aminoglycosides, Fluoroquinolones and Piperacillin-Tazobactam	All types of samples with deduplication	*Pseudomonas aeruginosa* resistant to one or two antimicrobials classes / Cephalosporins (0 months) *Pseudomonas aeruginosa* resistant to 3 or 4 antimicrobials classes / Meropenem (1 month)	UC
Zhang 2018	China	28 quarters	*Staphylococcus aureus*	All classes of antimicrobials	All types of samples with deduplication	MRSA */* Monobactam (2 quarters), Imidazole (1 quarter), sulfonamides (1 quarter) MRSA (negative correlation) / Glycopeptides (0 quarter), Oxazolidinone (2 quarters) aminoglycosides (2 quarters)	UC

Correlation between MRSA emergence and macrolide use 4 months previously, correlation between MRSA emergence and fluoroquinolone use 1 month previously

^2^ Multivariate model also including infection control practices such as consumption of hydroalcoholic solution and/or promotion of hand hygiene and/or screening for the carriage of multi-resistant bacteria

UK: United kingdom, MRSA: Methicillin-resistant Staphylococcus aureus, UC: Uncalculated, MDR: Multidrug resistant, NS: not studied, ESBL: Extended spectrum beta-lactamase, ERV: vancomycin-resistant Enterococcus

### Studies characteristics

The characteristics of the studies are summarized in Table 1.

The 28 included articles [8, 13–15, 21–44] were published from 2000 to 2023. The studies were mainly conducted in hospitals in the United Kingdom (5/28) [21, 23, 25, 34, 35], France (5/28) [13, 15, 22, 26, 42], Germany (4/28) [14, 28, 29, 33], China (3/28) [38, 43, 44] and Greece (3/28) [30, 31, 39]. Most articles studied the correlation between AMC and AMR month by month, with a median follow-up ranging from 15 to 174 months [24, 29].

Twenty-six studies [8, 14, 15, 21–38, 40–44] included all the bacteriological samples available, while one [13] focused on urine analyses and one [39] on blood cultures. When different samples from the same patient were positive for the same microorganism, samples were deduplicated in most studies (23/28) [8, 13–15, 21–23, 26–32, 34, 36–38, 40–44]. Studies most frequently focused on *Staphylococcus aureus* (9/28) [21–23, 29, 32, 34, 35, 41, 44], *Pseudomonas aeruginosa* (7/28) [8, 14, 24, 26, 27, 33, 40], *Klebsiella* sp. (7/28) [8, 25, 28, 38–40, 43] and *Escherichia coli* (6/28) [8, 15, 27, 28, 40] and on the resistance to cephalosporin, methicillin, carbapenem and fluoroquinolone. Only three studies focused on the emergence of MDR bacteria [14, 24, 26]. Only seven of the 28 studies focusing on hospital-acquired infections or colonization were restricted to samples taken after 48 h of hospital stay according to the definition of nosocomial infections [15, 21–23, 28, 29, 42].

Comprehensive monitoring of AMC was carried out in 12 of the 28 included studies [21–23, 28, 29, 32, 34, 35, 37, 41, 42, 44], while some studies specifically analyzed the effect of antimicrobial classes on the emergence of AMR: carbapenems (12/28) [8, 14, 15, 24, 26, 30, 31, 33, 36, 38, 40, 43], cephalosporins (12/28) [15, 26, 27, 30, 31, 33, 38, 40], fluoroquinolones (10/28) [13–15, 24, 26, 30, 31, 33, 36, 43] and piperacillin-tazobactam (8/28) [14, 15, 24, 30, 31, 33, 36, 43]. All studies reported at least one statistically significant correlation between antimicrobial use and AMR (Table 1).

### Correlation between antimicrobial consumption and resistance

The use of β-lactams was frequently reported to be correlated with the emergence of cephalosporin resistance. With a 0 to 5 months lag time, these correlations were reported for Enterobacterales and *P. aeruginosa* in seven and four studies, respectively and were mainly caused by extended-spectrum β-lactamases (ESBLs) and/or AmpC β-lactamases [8, 15, 24, 27, 28, 33, 36, 40, 42]. Overall, multivariate DR models including β-lactams and fluoroquinolones consumption explained 15–86% of variation in cephalosporin resistance [8, 15, 28, 36].

Three studies [15, 28, 42] reported specifically a correlation between fluoroquinolone consumption and cephalosporin resistance. The use of fluoroquinolones was also correlated with emergence of resistance to fluoroquinolone in Gram-negative bacteria with 0 to 5 months of lag time in six publications [13, 15, 24, 30, 42, 43]. In this case, DR model explained 40–66% of fluoroquinolone resistance variation [13, 15, 30, 43]. Fluoroquinolones use was also reported to be correlated with the emergence of methicillin resistant *S. aureus* (MRSA) in seven papers with a lag time of 2 to 4 months [21–23, 29, 34, 35, 41]. MRSA emergence was correlated with the use of macrolides in six articles, with a lag time ranging from 1 to 5 months. Multivariate DR model, including fluoroquinolone, macrolide and cephalosporin consumption, explained 41–96% of MRSA emergence [21, 22, 29, 34, 41].

Carbapenems use was correlated with the emergence of carbapenems resistance in *P. aeruginosa* in four studies [8, 24, 33, 40]. This correlation was also found in *Klebsiella pneumoniae* and *Acinetobacter baumanni*i in three and two papers, respectively [25, 30, 38, 40]. The lag time between emergence of carbapenems resistance and carbapenem consumption ranged between 0 and 12 months. DR models highlighted that 19 to 79% of carbapenem resistance rate variation was explained by carbapenem consumption [8, 25, 30, 38].

### Model description

Twenty-one studies (21/28) [8, 13–15, 21–23, 25, 26, 30, 31, 33–36, 38–43] used DR as described by Pankratz [11, 12]. In the seven remaining studies, the statistical models used, namely, “dynamic lag times series” and “multivariate ARIMA lag structure regression model” were assessed as a DR model (7/28) [24, 27–29, 32, 37, 44]. One study did not perform lag time correlation [27], see Table 1.

### Applications of DR model

Beyond correlating AMR and AMC, DR models have been reported to be useful to predict AMR emergence due to AMC [8, 14, 15, 27, 31, 34, 39]. They were considered effective tools for estimating the expected effect of a reduction in antimicrobial use on resistance [8, 14, 15, 22, 23, 28, 29, 34, 36, 41, 43]. Thus, they could be used to assess ASP efficiency and make new antimicrobial policies. However, their predictive capacity may be limited under certain conditions, notably when the incidence and prevalence of AMR is low [15].

### Quality of studies

**Fig. 2:**
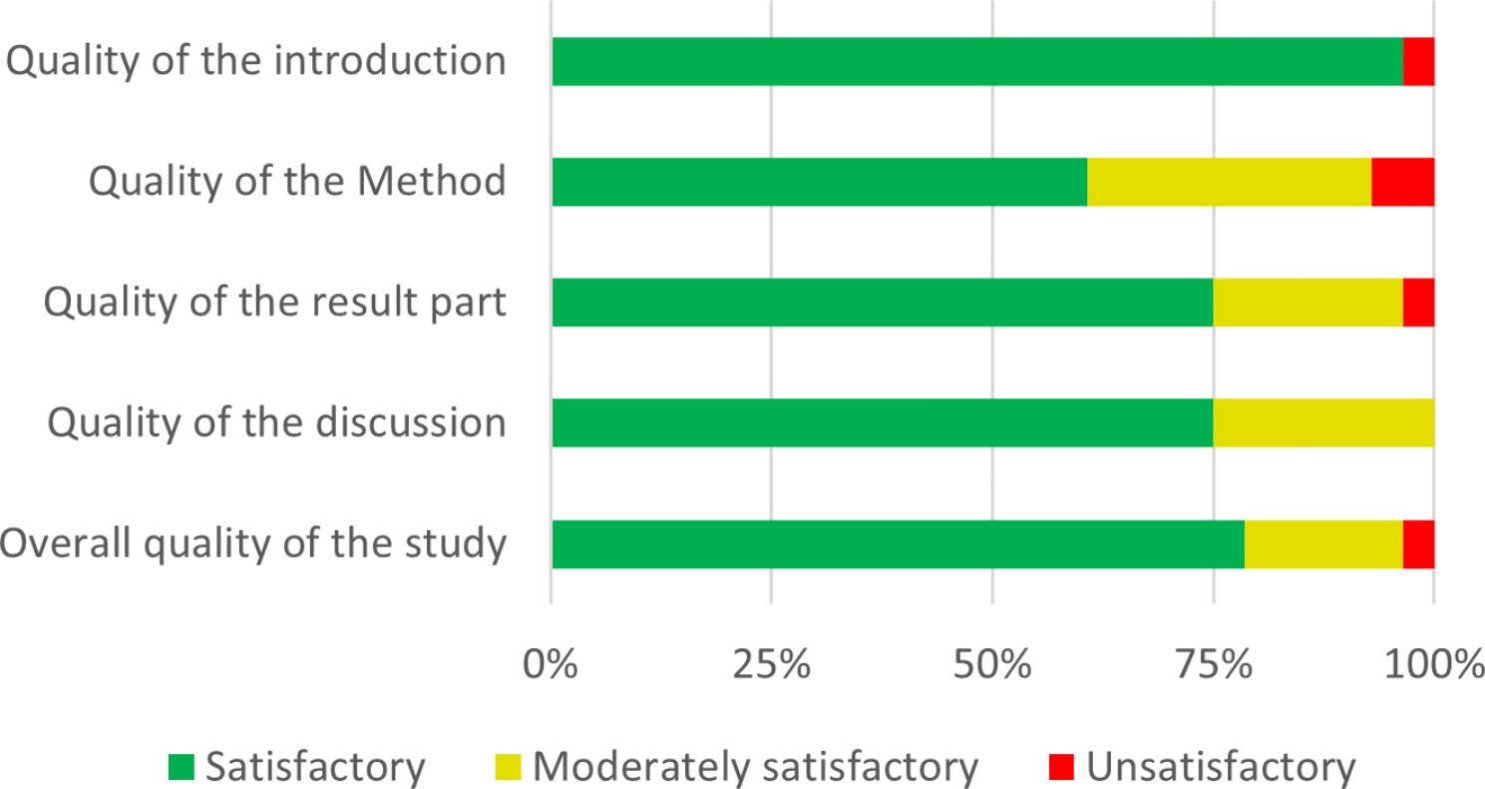
Risk of bias assessment in the overall studies

**Fig. 3:**
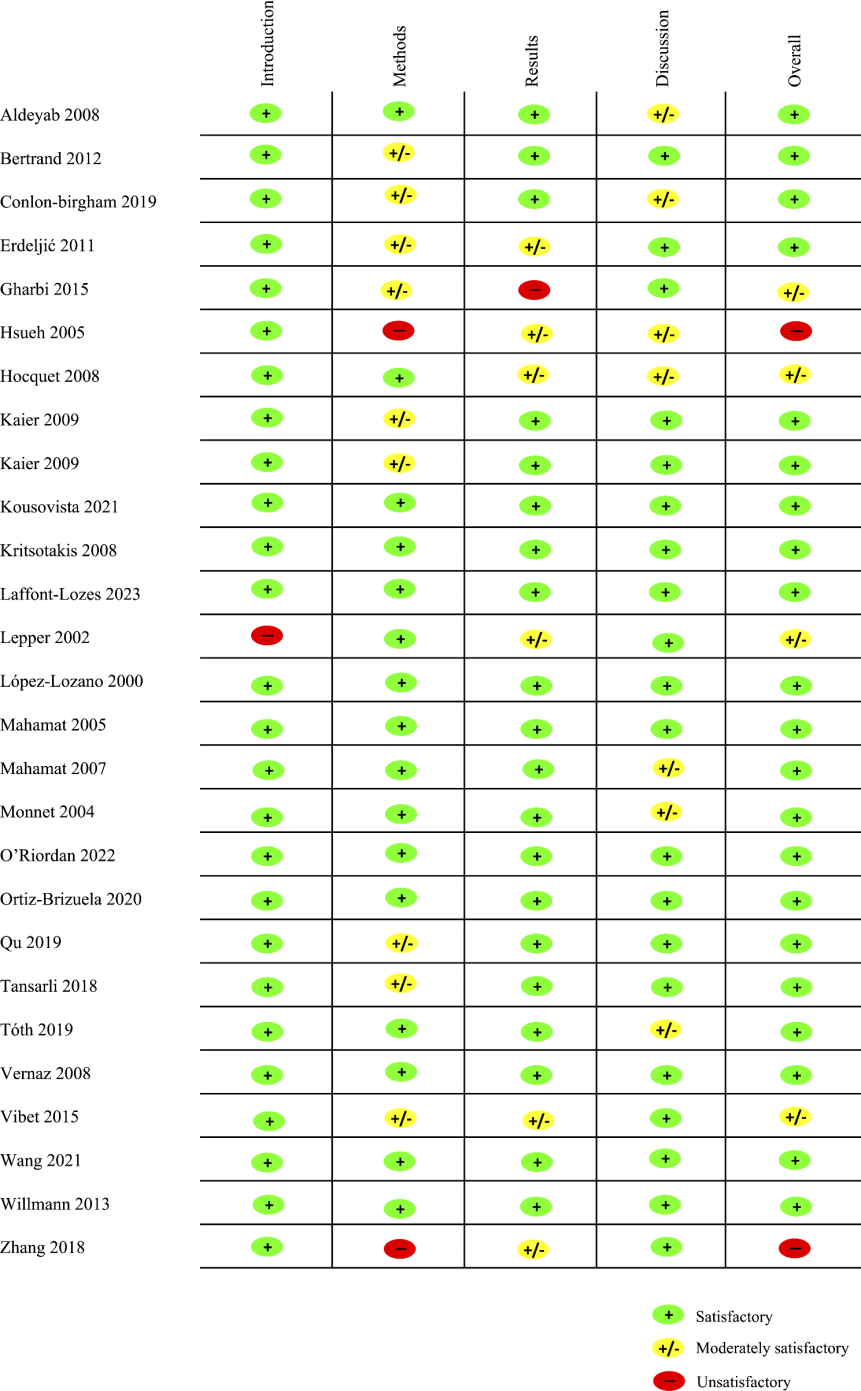
Risk of bias per study

The quality of studies was mainly satisfactory, except for two studies [27, 44] (Figs. 2 and 3). Two studies [23, 27] reported unclear methods, mentioning only the ARIMA model while both the ARIMA and LTF models are needed to perform a DR model. However, in numerous papers, the term ARIMA was used instead of DR [35, 37, 41], and studies reporting a dynamic relationship between AMR and AMC, suggested a DR model was used. Finally, results were not clearly reported in one studies [25] and the discussion of limitations was moderately satisfactory in seven articles [21, 23, 27, 28, 34, 35, 40].

## Discussion

In this review of 28 studies using DR models in hospital setting, we reported correlations between ß-lactams and fluoroquinolones use and cephalosporin resistance in Enterobacterales and *P. aeruginosa*, carbapenems use and carbapenem resistance in *P. aeruginosa, K. pneumoniae* and *A. baumannii*, and fluoroquinolones and macrolides use and methicillin resistance in *S. aureus*. A lag time of 2 to 4 months between AMC and the emergence of AMR was reported in most studies. In multivariate DR models, the burden of AMC on AMR fluctuation ranged from 15 to 96%. We also reported the potential usefulness of DR models in AMR prediction and, in accordance, their possible value in guiding ASP.

Many studies using linear or logistic regression models [45–47] or interrupted time series [48–51] have highlighted the selection pressure of antimicrobials. Indeed, antimicrobials with broad-spectrum activity, are frequently reported to be associated with AMR emergence, especially in the ESKAPE group (namely, *Enterococcus faecium*, *S. aureus*, *K. pneumoniae*, *A. baumannii*, *P. aeruginosa* and *Enterobacter spp*) [49, 52–57]. These findings are in line with those reported using the DR models, which however have advantages over other studies [10, 58, 59]. Firstly, they are based on big data analyses providing a comprehensive view of selection pressure [7]. Secondly, the LFT allows temporal correlation, which increased the model’s ability to early detect a correlation between antimicrobial use and AMR emergence as reported by Erdeljić et al. [24]. Thirdly, since AMR rates are not independent series, DR models avoid the bias of overestimating correlation, since they take into account the autocorrelation of the series studied [10, 53]. Finally, multivariate analysis of DR models could explain the burden of AMC in resistance fluctuation relative to the colonization pressure [10, 14].

Using DR models for assessing the correlation between AMC and AMR, the coefficients of determination represent the burden of a class of antibiotic on AMR [8]. It should be noted that coefficients of determination reported in our systematic review are disparate. This may be explained by the heterogeneity of the studies included, notably due to variations in practices such as hygiene protocols, variations in the bacterial species involved or stochastic variations [23, 24, 34, 53]. The crucial role of infection control procedures especially hand hygiene, and the strategic application of measures designed to curtail microbial transmission in influencing the landscape of AMR must be emphasized here, as they modify the complex relationship between AMC and AMR [60]. In addition, the genetic mechanisms underlying AMR (mutations and acquisition of resistance genes) lead to the development of cross-resistance - where AMC of one class results in resistance to other classes - contributing to the emergence of MDR strains [61]. Thus, the study of the correlation between AMC and AMR is complicated by the interplay of these genetic factors, which vary between antibiotic classes and bacterial species [62]. Moreover, DR models are based on large dataset analyses [7, 11], in which individual risk factors for AMR, such as age, immunosuppression, or chronic diseases, dose and duration of antimicrobial therapy, are not included. Nevertheless, it should be highlighted that antibiotic classes known for their ability to induce AMR, such as fluoroquinolones, exhibit both high correlation coefficients and narrow confidence intervals. This underscores their significant impact on the emergence of AMR [13, 15, 30, 43].

Finally, compared with other models, DR models have the advantage of being able to predict future fluctuations in AMR as a function of AMC. With the growing clinical importance of mathematical models to guide antimicrobials selection [63], DR models are seen as promising tools for the development of new ASPs. In fact, they can help select the most interesting antimicrobial agent to target, and then measure the effects of a given ASP on the incidence of AMR compared with the incidence initially predicted [8, 14, 31]. As predictive tools, they could also be used to visualize the effect of a theoretical decrease in AMC on future AMR incidence [14]. However, external validations and cross validations between observations and predictions are mandatory, all the more so as the predictive capabilities of models can be undermined when the incidence of AMR is low [14, 15].

We must acknowledge some limitations in this review. First, studies were mainly geographically limited to European and Asian countries, limiting the generalization of the results to other geographical areas with different bacterial epidemiology. Second, studies included in this report have methodological limitations that could have induced bias in result interpretation. Particularly, several studies did not exclude duplicate samples (5 of 28) or patients previously known to be carriers of MDR bacteria [24, 25, 33, 35, 39]. Some studies were based on short follow-up times for DR modeling and unclear sampling timing [24, 27, 28, 30, 33, 36, 44], and three-quarters of included articles did not exclude samples taken within the first 48 h of hospitalization [8, 13, 14, 24, 25, 27, 30–41, 43, 44, 64]. Last, DR models focusing only on selection pressure and did not include the burden of colonization pressure. Nonetheless, these biases are common and inherent to all time series studies, which remain a reference model for studying AMR fluctuations at hospital scale.

## Conclusion

To our knowledge, this report is the first systematic review of the use of DR models to assess the correlation between AMC and AMR at the hospital level. Although statistically recognized as a valid model for studying the correlation between AMC and AMR, DR models are little used in the literature. They have been used to highlight the correlation between cephalosporins use and cephalosporin resistance in *E. coli* and *P. aeruginosa*, carbapenems use and carbapenem resistance in *P. aeruginosa* and *A. baumannii* and fluoroquinolone use and fluoroquinolone resistance in Gram-negative bacteria and MRSA, after a lag-time of 2 to 4 months. Further studies are mandatory to analyze the link between AMR in other bacterial species and antimicrobials, and to assess the use of DR models for AMR forecasting and for ASPs building and evaluation.

### Electronic supplementary material

Below is the link to the electronic supplementary material.

**Supplementary Data: Table S1**

## Data Availability

The authors consent to share the collected data with others. Data will be available without undue reservation, immediately after the main publication and indefinitely.

## List of abbreviations

AMC: Antimicrobial consumption
AMR: Antimicrobial resistance
ARIMA: Autoregressive integrated moving average
ASPs: Antimicrobials stewardship program
DR: Dynamic regression
ESBLs: Extended-spectrum β-lactamases
LTF: Linear transfer function
MDR: Multidrug resistance
MRSA: Methicillin resistant *S. aureus*
WHO: World health organization

## References

[1] Carlet J, Collignon P, Goldmann D, Goossens H, Gyssens IC, Harbarth S (2011). Society’s failure to protect a precious resource: antibiotics. The Lancet.

[2] Laxminarayan R, Duse A, Wattal C, Zaidi AKM, Wertheim HFL, Sumpradit N (2013). Antibiotic resistance—the need for global solutions. Lancet Infect Dis.

[3] O’Neill J (2014). Review on Antimicrobial Resistance.

[4] Global antimicrobial resistance surveillance (2020). System (GLASS) report: early implementation 2020.

[5] Barlam TF, Cosgrove SE, Abbo LM, MacDougall C, Schuetz AN, Septimus EJ (2016). Implementing an antibiotic stewardship program: guidelines by the infectious Diseases Society of America and the Society for Healthcare Epidemiology of America. Clin Infect Dis.

[6] Antimicrobial stewardship.: Systems and processes for effective antimicrobial medicine use. NICE Guidel. 2015. 10.1093/jacamr/dlz025 PMC8210248 34222900

[7] Eliopoulos GM, Shardell M, Harris AD, El-Kamary SS, Furuno JP, Miller RR (2007). Statistical analysis and application of quasi experiments to Antimicrobial Resistance intervention studies. Clin Infect Dis.

[8] López-Lozano J-M, Monnet DL, Yagüe A, Burgos A, Gonzalo N, Campillos P (2000). Modelling and forecasting antimicrobial resistance and its dynamic relationship to antimicrobial use: a time series analysis. Int J Antimicrob Agents.

[9] Monnet DL, López-Lozano J-M, Campillos P, Burgos A, Yagüe A, Gonzalo N (2001). Making sense of antimicrobial use and resistance surveillance data: application of ARIMA and transfer function models. Clin Microbiol Infect.

[10] Mahamat A, Daurès JP, Sotto A (2005). Évaluation de la relation consommation de fluoroquinolones et émergence de résistance chez Escherichia coli: rôles respectif et comparatif des études observationnelles et quasi expérimentales. Médecine Mal Infect.

[11] Box GEP, Jenkins GM. Time series analysis: forecasting and control.San Francisco, CA: Holden-Day. (1970) 1976. 575 p. 1970;1.

[12] Pankratz A (1991). Forecasting with dynamic regression models.

[13] Mahamat A, Lavigne JP, Fabbro-Peray P, Kinowski JM, Daurès JP, Sotto A (2005). Evolution of fluoroquinolone resistance among Escherichia coli urinary tract isolates from a french university hospital: application of the dynamic regression model. Clin Microbiol Infect.

[14] Willmann M, Marschal M, Hölzl F, Schröppel K, Autenrieth IB, Peter S (2013). Time Series Analysis as a Tool to predict the impact of Antimicrobial Restriction in Antibiotic Stewardship Programs using the Example of Multidrug-Resistant Pseudomonas aeruginosa. Antimicrob Agents Chemother.

[15] Laffont-Lozes P, Salipante F, Leguelinel-Blache G, Dunyach-Remy C, Lavigne J-P, Sotto A (2023). Effect of antimicrobial consumption on Escherichia coli resistance: assessment and forecasting using dynamic regression models in a french university hospital (2014–2019). Int J Antimicrob Agents.

[16] Moher D, Liverati A, Tetzlaff J, Altman DG, the PRISMA Group. Preferred reporting items for systematic reviews and Meta-analyses: the PRISMA Statement. PLoS Med. 2009;6. PMC3090117 21603045

[17] All the Confusion about ARIMA, Transfer Function ARIMAX. Dynamic Regression Models [Internet]. Ruqin Ren. 2020 [cited 2022 Feb 4]. Available from: https://ruqinren.wordpress.com/2020/02/21/all-the-confusion-about-arima-arimax-transfer-function-dynamic-regression-models/.

[18] Québec University Hospitals. GRILLE, D’ÉVALUATION DE LA, QUALITÉ DES ÉTUDES Étude observationnelle [Internet]. Available from: www.chudequebec.ca/chudequebec.ca/files/8f/8f479c9b-c23d-465a-83dc-f80bdc3734f3.pdf?msclkid=f5c5f19acbab11eca90208e423e81091.

[19] Wells GA, Shea B, O’Connell D, Paterson J, Welch V, Losos M et al. The Newcastle-Ottawa Scale (NOS) for assessing the quality of nonrandomised studies in meta-analyses. 2014.

[20] von Elm E, Altman DG, Egger M, Pocock SJ, Gøtzsche PC, Vandenbroucke JP (2008). The strengthening the reporting of Observational Studies in Epidemiology (STROBE) statement: guidelines for reporting observational studies. J Clin Epidemiol.

[21] Aldeyab MA, Monnet DL, Lopez-Lozano JM, Hughes CM, Scott MG, Kearney MP (2008). Modelling the impact of antibiotic use and infection control practices on the incidence of hospital-acquired methicillin-resistant Staphylococcus aureus: a time-series analysis. J Antimicrob Chemother.

[22] Bertrand X, Lopez-Lozano JM, Slekovec C, Thouverez M, Hocquet D, Talon D (2012). Temporal effects of infection control practices and the use of antibiotics on the incidence of MRSA. J Hosp Infect.

[23] Conlon-Bingham GM, Aldeyab M, Scott M, Kearney MP, Farren D, Gilmore F (2019). Effects of Antibiotic Cycling Policy on incidence of Healthcare-Associated MRSA and *Clostridioides difficile* infection in secondary Healthcare settings. Emerg Infect Dis.

[24] Erdeljić V, Francetić I, Bošnjak Z, Budimir A, Kalenić S, Bielen L (2011). Distributed lags time series analysis versus linear correlation analysis (Pearson’s r) in identifying the relationship between antipseudomonal antibiotic consumption and the susceptibility of Pseudomonas aeruginosa isolates in a single Intensive Care Unit of a tertiary hospital. Int J Antimicrob Agents.

[25] Gharbi M, Moore LSP, Gilchrist M, Thomas CP, Bamford K, Brannigan ET (2015). Forecasting carbapenem resistance from antimicrobial consumption surveillance: Lessons learnt from an OXA-48-producing Klebsiella pneumoniae outbreak in a West London renal unit. Int J Antimicrob Agents.

[26] Hocquet D, Muller A, Blanc K, Plésiat P, Talon D, Monnet DL (2008). Relationship between antibiotic use and incidence of MexXY-OprM overproducers among clinical Isolates of Pseudomonas aeruginosa. Antimicrob Agents Chemother.

[27] Hsueh P-R, Chen W-H, Luh K-T (2005). Relationships between antimicrobial use and antimicrobial resistance in Gram-negative bacteria causing nosocomial infections from 1991–2003 at a university hospital in Taiwan. Int J Antimicrob Agents.

[28] Kaier K, Frank U, Hagist C, Conrad A, Meyer E (2009). The impact of antimicrobial drug consumption and alcohol-based hand rub use on the emergence and spread of extended-spectrum -lactamase-producing strains: a time-series analysis. J Antimicrob Chemother.

[29] Kaier K, Hagist C, Frank U, Conrad A, Meyer E (2009). Two time-series analyses of the impact of antibiotic consumption and alcohol-based Hand Disinfection on the Incidences of nosocomial methicillin-resistant *Staphylococcus aureus* infection and *Clostridium difficile* infection. Infect Control Hosp Epidemiol.

[30] Kousovista R, Athanasiou C, Liaskonis K, Ivopoulou O, Ismailos G, Karalis V (2021). Correlation between Acinetobacter baumannii Resistance and Hospital Use of Meropenem, Cefepime, and ciprofloxacin: Time Series Analysis and dynamic regression models. Pathogens.

[31] Kritsotakis EI, Christidou A, Roumbelaki M, Tselentis Y, Gikas A (2008). The dynamic relationship between antibiotic use and the incidence of vancomycin-resistant Enterococcus: time-series modelling of 7-year surveillance data in a tertiary-care hospital. Clin Microbiol Infect.

[32] Lee Y-T, Chen S-C, Lee M-C, Hung H-C, Huang H-J, Lin H-C (2012). Time-series analysis of the relationship of antimicrobial use and hand hygiene promotion with the incidence of healthcare-associated infections. J Antibiot (Tokyo).

[33] Lepper P, Grusa E, Reichl H, Högel J, Trautmann M (2002). Consumption of Imipenem correlates with β-Lactam resistance in Pseudomonas aeruginosa. Antimicrob Agents Chemother.

[34] Mahamat A, MacKenzie FM, Brooker K, Monnet DL, Daures JP, Gould IM (2007). Impact of infection control interventions and antibiotic use on hospital MRSA: a multivariate interrupted time-series analysis. Int J Antimicrob Agents.

[35] Monnet DL, MacKenzie FM, López-Lozano JM, Beyaert A, Camacho M, Wilson R (2004). Antimicrobial Drug Use and Methicillin-resistant *Staphylococcus aureus*, Aberdeen, 1996–2000. Emerg Infect Dis.

[36] O’Riordan F, Shiely F, Byrne S, O’Brien D, Ronayne A, Fleming A (2022). Antimicrobial use and antimicrobial resistance in Enterobacterales and Enterococcus faecium: a time series analysis. J Hosp Infect.

[37] Ortiz-Brizuela E, Caro-Vega Y, Bobadilla-del-Valle M, Leal-Vega F, Criollo-Mora E, López Luis BA (2020). The influence of hospital antimicrobial use on carbapenem-non-susceptible Enterobacterales incidence rates according to their mechanism of resistance: a time-series analysis. J Hosp Infect.

[38] Qu X, Wang H, Chen C, Tao Z, Yin C, Yin A (2019). Surveillance of carbapenem-resistant Klebsiella pneumoniae in chinese hospitals - a five-year retrospective study. J Infect Dev Ctries.

[39] Tansarli GS, Papaparaskevas J, Balaska M, Samarkos M, Pantazatou A, Markogiannakis A (2018). Colistin resistance in carbapenemase-producing Klebsiella pneumoniae bloodstream isolates: evolution over 15 years and temporal association with colistin use by time series analysis. Int J Antimicrob Agents.

[40] Tóth H, Fésűs A, Kungler-Gorácz O, Balázs B, Majoros L, Szarka K (2019). Utilization of Vector Autoregressive and Linear transfer models to follow up the antibiotic resistance spiral in Gram-negative Bacteria from cephalosporin consumption to Colistin Resistance. Clin Infect Dis.

[41] Vernaz N, Sax H, Pittet D, Bonnabry P, Schrenzel J, Harbarth S (2008). Temporal effects of antibiotic use and hand rub consumption on the incidence of MRSA and Clostridium difficile. J Antimicrob Chemother.

[42] Vibet M-A, Roux J, Montassier E, Corvec S, Juvin M-E, Ngohou C (2015). Systematic analysis of the relationship between antibiotic use and extended-spectrum beta-lactamase resistance in Enterobacteriaceae in a french hospital: a time series analysis. Eur J Clin Microbiol Infect Dis.

[43] Wang Y, Zhong H, Han X, Wang N, Cai Y, Wang H (2021). Impact of antibiotic prescription on the resistance of Klebsiella pneumoniae at a tertiary hospital in China, 2012–2019. Am J Infect Control.

[44] Zhang D, Cui K, Wang T, Dong H, Feng W, Ma C (2019). Trends in and correlations between antibiotic consumption and resistance of *Staphylococcus aureus* at a tertiary hospital in China before and after introduction of an antimicrobial stewardship programme. Epidemiol Infect.

[45] Ruiz J, Gordon M, Villarreal E, Frasquet J, Sánchez M, Martín M (2019). Influence of antibiotic pressure on multi-drug resistant Klebsiella pneumoniae colonisation in critically ill patients. Antimicrob Resist Infect Control.

[46] Quan J, Zhao D, Liu L, Chen Y, Zhou J, Jiang Y (2017). High prevalence of ESBL-producing Escherichia coli and Klebsiella pneumoniae in community-onset bloodstream infections in China. J Antimicrob Chemother.

[47] Brosh-Nissimov T, Navon-Venezia S, Keller N, Amit S (2019). Risk analysis of antimicrobial resistance in outpatient urinary tract infections of young healthy adults. J Antimicrob Chemother.

[48] Boel J, Andreasen V, Jarløv JO, Østergaard C, Gjørup I, Bøggild N (2016). Impact of antibiotic restriction on resistance levels of *Escherichia coli*: a controlled interrupted time series study of a hospital-wide antibiotic stewardship programme. J Antimicrob Chemother.

[49] Bosso JA, Mauldin PD (2006). Using interrupted Time Series Analysis to assess Associations of Fluoroquinolone Formulary Changes with susceptibility of Gram-Negative pathogens and isolation rates of Methicillin-Resistant Staphylococcus aureus. Antimicrob Agents Chemother.

[50] Church EC, Mauldin PD, Bosso JA (2011). Antibiotic resistance in *Pseudomonas aeruginosa* Related to Quinolone Formulary Changes: an interrupted Time Series Analysis. Infect Control Hosp Epidemiol.

[51] Davey P, Marwick CA, Scott CL, Charani E, McNeil K, Brown E et al. Interventions to improve antibiotic prescribing practices for hospital inpatients. Cochrane Database Syst Rev. 2017. 10.1002/14651858.CD003543.pub4 PMC6464541 28178770

[52] De Oliveira DMP, Forde BM, Kidd TJ, Harris PNA, Schembri MA, Beatson SA (2020). Antimicrobial Resistance in ESKAPE Pathogens. Clin Microbiol Rev.

[53] Charbonneau P, Parienti J-J, Thibon P, Ramakers M, Daubin C, du Cheyron D (2006). Fluoroquinolone Use and Methicillin-Resistant Staphylococcus aureus isolation rates in hospitalized patients: a quasi experimental study. Clin Infect Dis.

[54] Borde JP, Kern WV, Hug M, Steib-Bauert M, de With K, Busch H-J (2015). Implementation of an intensified antibiotic stewardship programme targeting third-generation cephalosporin and fluoroquinolone use in an emergency medicine department. Emerg Med J.

[55] Lewis GJ, Fang X, Gooch M, Cook PP (2012). Decreased resistance of *Pseudomonas aeruginosa* with Restriction of Ciprofloxacin in a large Teaching Hospital’s Intensive Care and Intermediate Care Units. Infect Control Hosp Epidemiol.

[56] Lafaurie M, Porcher R, Donay J-L, Touratier S, Molina J-M (2012). Reduction of fluoroquinolone use is associated with a decrease in methicillin-resistant Staphylococcus aureus and fluoroquinolone-resistant Pseudomonas aeruginosa isolation rates: a 10 year study. J Antimicrob Chemother.

[57] Sarma JB, Marshall B, Cleeve V, Tate D, Oswald T, Woolfrey S (2015). Effects of fluoroquinolone restriction (from 2007 to 2012) on resistance in Enterobacteriaceae: interrupted time-series analysis. J Hosp Infect.

[58] Harbarth S, Harris AD, Carmeli Y, Samore MH (2001). Parallel analysis of individual and aggregated data on antibiotic exposure and resistance in Gram-Negative Bacilli. Clin Infect Dis.

[59] Harris AD, Karchmer TB, Carmeli Y, Samore MH (2001). Methodological Principles of Case-Control Studies that analyzed risk factors for Antibiotic Resistance: a systematic review. Clin Infect Dis.

[60] Allegranzi B, Pittet D (2009). Role of hand hygiene in healthcare-associated infection prevention. J Hosp Infect.

[61] Laxminarayan R, Matsoso P, Pant S, Brower C, Røttingen J-A, Klugman K (2016). Access to effective antimicrobials: a worldwide challenge. The Lancet.

[62] Wright GD (2007). The antibiotic resistome: the nexus of chemical and genetic diversity. Nat Rev Microbiol.

[63] Sotto A, Lavigne J-P (2012). A mathematical model to guide antibiotic treatment strategies. BMC Med.

[64] Hocquet D, Muller A, Blanc K, Plésiat P, Talon D, Monnet DL (2008). Relationship between antibiotic use and incidence of MexXY-OprM overproducers among clinical Isolates of *Pseudomonas aeruginosa*. Antimicrob Agents Chemother.

