# Correction: Usefulness of dynamic regression time series models for studying the relationship between antimicrobial consumption and bacterial antimicrobial resistance in hospitals: a systematic review

**DOI:** 10.1186/s13756-024-01387-4

**Published:** 2024-03-21

**Authors:** Paul Laffont-Lozes, Romaric Larcher, Florian Salipante, Geraldine Leguelinel-Blache, Catherine Dunyach-Remy, Jean-Philippe Lavigne, Albert Sotto, Paul Loubet

**Affiliations:** 1 Department of Pharmacy, Nimes University Hospital, Nimes, France; 2 Infectious and Tropical Diseases Department, Nimes University Hospital, Nimes, France; 3 PhyMedExp, INSERM U1046, CNRS, University of Montpellier, Montpellier, France; 4 Department of Biostatistics, Epidemiology, Public Health, and Innovation in Methodology (BESPIM), University of Montpellier, Nîmes University Hospital, Nimes, France; 5 Department of Microbiology and Hospital Hygiene, Nimes University Hospital, Nimes, France; 6 VBIC, INSERM U1047, University of Montpellier, Nimes, France; 7 Service des Maladies Infectieuses et Tropicales, Hôpital Caremeau – CHU de Nimes, 1 Place Robert Debre, Nîmes, 30000 France

**Correction: Antimicrobial Resistance & Infection Control (2023) 12:100** 10.1186/s13756-023-01302-3

The original article mistakenly omitted all rows below the 8th row of Table 1.

The table has since been amended to show the entirety of the table as was initially intended.

