# Supplementary material for: Usefulness of dynamic regression time series models for studying the relationship between antimicrobial consumption and bacterial antimicrobial resistance in hospitals: a systematic review
Source: Antimicrob Resist Infect Control. 2023 Sep 12;12:100. doi: 10.1186/s13756-023-01302-3 (PMC10496333; doi:10.1186/s13756-023-01302-3)
Supplement: Supplementary file 1 — Supplementary Data: Table S1 [file 13756_2023_1302_MOESM1_ESM.doc]

**Supplementary Data**

**Table S1**

| **Author(s)** |  | **publication** |  |
| --- | --- | --- | --- |
| **Purpose of the Study** |  | | |
| **Study type** | Interventional □ No interventional □ | | |
| **Assessor** |  | **Date** |  |

| **Introduction** | | | |
| --- | --- | --- | --- |
| I1 | Is the objective of the article stated by specifying, at a minimum, the intervention and data studied (if applicable) ? | Yes □ | No □ |
| I2 | Overall Quality of the introduction part | Satisfactory □ | Unsatisfactory □ |

| **Method** | | | | | |
| --- | --- | --- | --- | --- | --- |
| M1 | Is the context of the study (location, period of data collection) clearly described? | Yes □ | | No □ | |
| M2 | Are the inclusion/exclusion criteria for the data to be collected specified? | Yes □ | | No □ | |
| M3 | Is the data collection method described and adequate? | Yes □ | | No □ | |
| M4 | Is the intervention sufficiently described? | Yes □ | No □ | | N.A □ |
| M5 | Is the comparator sufficiently described? | Yes □ | No □ | | N.A □ |
| M6 | Are all measurement tools used standardized, valid and reliable? | Yes □ | | No □ | |
| M7 | Is the statistical time series model used detailed | Yes □ | | No □ | |
| M8 | Overall Quality of the Method part | Satisfactory □ | | Unsatisfactory □ | |

| **Results** | | | | | |
| --- | --- | --- | --- | --- | --- |
| R1 | Are the data studied in line with the objectives? | Yes □ | | No □ | |
| R2 | Is the amount of data analyzed sufficient to obtain usable results with the statistical model ? | Yes □ | | No □ | |
| R3 | Is the number of observations (pooled data) analyzed sufficient to obtain usable results with statistical model? | Yes □ | No □ | | N.A □ |
| R4 | Is the detail of the data collection sufficiently described? | Yes □ | | No □ | |
| R5 | Do the results presented take into account potentially confounding factors? | Yes □ | | No □ | |
| R6 | Are the analyses performed as planned? | Yes □ | | No □ | |
| R7 | Is the expression of results clear and detailed? | Yes □ | | No □ | |
| R8 | Overall Quality of the Result part | Satisfactory □ | | Unsatisfactory □ | |

| **Discussion** | | | |
| --- | --- | --- | --- |
| D1 | Do the study findings address the primary objectives? | Yes □ | No □ |
| D2 | Do the authors identify the limitations of the study? | Yes □ | No □ |
| D3 | Is the consistency of the results with other studies discussed? | Yes □ | No □ |
| D4 | Are the study findings consistent with the key findings? | Yes □ | No □ |
| D5 | Overall Quality of the disucssion part | Satisfactory □ | Unsatisfactory □ |

| Overall assessment of the quality of the study | | |
| --- | --- | --- |
| The overall quality of the study is | Satisfactory □ | Unsatisfactory □ |

According to GRILLE D’ÉVALUATION DE LA QUALITÉ DES ÉTUDES Étude observationnelle de CHU de Québec ([8f479c9b-c23d-465a-83dc-f80bdc3734f3.pdf (chudequebec.ca)](https://www.chudequebec.ca/chudequebec.ca/files/8f/8f479c9b-c23d-465a-83dc-f80bdc3734f3.pdf)), COMPUS Adapted SIGN 50 [(http://www.sign.ac.uk/methodology/checklists.html)](http://www.sign.ac.uk/methodology/checklists.html), Downs and Black scale (1998) (Downs et Black, 1998), STROBE list (von Elm et al. 2008), Newcastle-Ottawa scale corhort studies [(http://www.ohri.ca/programs/clinical_epidemiology/nos_manual.pdf)](../(http://www.ohri.ca/programs/clinical_epidemiology/nos_manual.pdf)), Report of the IPSOR Task Force on retrospective databases (Montheral et al. 2003), CASP tool (Critical Appraisal Skills Programme, and l’Agency for Healthcare Research and Quality (AHRQ 2013).

# Références

- *Critical Appraisal Skills Programme* (*CAPS*). 12 questions pour interpréter les études de cohorte. Traduction libre de l’Institut national d’excellence en santé et services sociaux (INESSS) du Québec, 2015. Disponible à <http://www.inesss.qc.ca/fileadmin/doc/INESSS/DocuMetho/CASP_cohorte_FR2013_V14012015.pdf>
- Downs, S.H., Black, N. The feasibility of creating a checklist for the assessment of the methodological quality both of randomized and non-randomized studies of health care interventions. Journal of Epidemiology Community Health, 1998, 52, 377-384.
- Montheral, B., Brooks, J., Clark, M, Crown, W.H., Davey, P., Hutchins, D., Martin, B.C., Stang, P.A checklist for retrospective database studies – Report of the IPSOR Task Force on retrospective databases. Value in Health, 2003, 6(2), 90-97.
- Viswanathan M, Berkman ND, Dryden DM, Hartling L. Assessing Risk of Bias and Confounding in Observational Studies of Interventions or Exposures: Further Development of the RTI Item Bank. Methods Research Report. (Prepared by RTI–UNC Evidence-based Practice Center under Contract No. 290- 2007-10056-I). AHRQ Publication No. 13-EHC106-EF. Rockville, MD: Agency for Healthcare Research and Quality; August 2013. [www.effectivehealthcare.ahrq.gov/reports/final.cfm.](http://www.effectivehealthcare.ahrq.gov/reports/final.cfm)
- von Elm E, Altman DG, Egger M, Pocock SJ, Gøtzsche PC, Vandenbroucke JP; STROBE Initiative. The Strengthening the Reporting of Observational Studies in epidemiology (STROBE) statement: guidelines for reporting observational studies. J Clin Epidemiol. 2008 Apr;61(4):344-9.
